# Are We Slaves or Freemen?

**Published:** 1882-05

**Authors:** 


Are we Slaves or Freemen?—This is the startling question asked by Dr. E. A. Cobleigh in the Cincinnati *Medical News*. We should not like to answer the question unless it is made a little more definite. A doctor may be a slave to work, or to study, or to the desire of money-getting, or he may even be a slave to indolence. In fact, if most of the complaints which come to our ears are true, the last is probably the form of slavery from which most young practitioners would like to escape. A physician who has established a practice sufficient to keep him moderately busy, is generally satisfied with the equivalent which he receives for his labor. One who is overworked can easily cut down the demands upon his time by placing an increased value upon it. Of course, many disagreeable things occur in a general practice, and many discomforts must be submitted to, but we venture to express the opinion that those who are unwilling to submit to these things as cheerfully as is consistent with the circumstances, have probably mistaken their calling. The practice of medicine ought to be made a "business" perhaps quite as much as a "charity," but it should and can be done in a dignified way. The public is apt to pay us for our services at our own valuation, and it is easy enough to get rid of a non-paying clientele by declining to add to an already too large bill when a subsequent sickness in the family of the debtor calls for renewed visits. We are old-fashioned enough, however, to hope that those who practice medicine merely "for the cold, sordid, base and worldly lucre" are a minority in the ranks of the profession.

"The serfdom of our calling" is what most of us expect when we enter the profession. If one would rather go to the theatre, "the social gathering, or even the sanctuary" than perform the duty of humanity which he has, in form at least, made oath to do, it would be better for him, the profession, and perhaps his patients also if he were to seek some other business in which the demands on his charity would be less frequent and burdensome.

It has been our experience and the result of considerable close observation, that a manly independence will secure to every one—even to a physician—the respect of the community in which he lives. The physician who permits any client to assail him with "a pyrotechnic display of profanity" for refusing to be imposed upon, has probably brought this treatment on himself by previously neglecting to exact that respect to which his position entitles him. We decline to believe the assertion of Dr. Cobleigh that physicians are generally imposed upon or "made slaves" of by their patients. Our observation and experience have been otherwise.

